# Associations of context-specific sitting time with markers of cardiometabolic risk in Australian adults

**DOI:** 10.1186/s12966-018-0748-3

**Published:** 2018-11-20

**Authors:** Paddy C. Dempsey, Nyssa T. Hadgraft, Elisabeth A. H. Winkler, Bronwyn K. Clark, Matthew P. Buman, Paul A. Gardiner, Neville Owen, Brigid M. Lynch, David W. Dunstan

**Affiliations:** 1 Physical Activity, Metabolic & Vascular Physiology and Behavioural Epidemiology Laboratories, Baker Heart and Diabetes Institute, Level 4, 99 Commercial Rd, Melbourne, VIC 3004 Australia; 2 Centre for Urban Transitions, Swinburne University of Technology, Melbourne, VIC Australia; 3 MRC Epidemiology Unit, Institute of Metabolic Science, University of Cambridge, Cambridge Biomedical Campus, Cambridge, UK; 4 School of Public Health/Centre for Health Services Research, The University of Queensland, Brisbane, QLD Australia; 5 College of Health Solutions, Arizona State University, 550 N 3rd Street, Phoenix, AZ 85004 USA; 6 Melbourne School of Population and Global Health, The University of Melbourne, Melbourne, VIC Australia; 7 Central Clinical School/Department of Epidemiology and Preventive Medicine, Faculty of Medicine, Nursing and Health Sciences, Monash University, Melbourne, VIC Australia; 8 Cancer Epidemiology and Intelligence Division, Cancer Council Victoria, Melbourne, VIC 3004 Australia; 9 Institute of Physical Activity and Nutrition Research, School of Exercise and Nutrition Sciences, Deakin University, Melbourne, VIC Australia; 10 Mary MacKillop Institute of Health Research, Australian Catholic University, Melbourne, VIC Australia; 11 School of Sport Science, Exercise and Health, The University of Western Australia, Perth, WA Australia

**Keywords:** Sedentary behaviour, Sitting, Transport sitting, Occupational sitting, Television viewing, Computer, Diabetes, Cardiovascular disease, Adiposity, Cardiometabolic

## Abstract

**Background:**

High volumes of sitting time are associated with an elevated risk of type 2 diabetes and cardiovascular disease, and with adverse cardiometabolic risk profiles. However, previous studies have predominately evaluated only total sitting or television (TV) viewing time, limiting inferences about the specific cardiometabolic health impacts of sitting accumulated in different contexts. We examined associations of sitting time in four contexts with cardiometabolic risk biomarkers in Australian adults.

**Methods:**

Participants (*n* = 3429; mean ± SD age 58 ± 10 years) were adults without clinically diagnosed diabetes or cardiovascular disease from the 2011–2012 Australian Diabetes, Obesity and Lifestyle (AusDiab) study. Multiple linear regressions examined associations of self-reported context-specific sitting time (occupational, transportation, TV-viewing and leisure-time computer use) with a clustered cardiometabolic risk score (CMR) and with individual cardiometabolic risk biomarkers (waist circumference, BMI, resting blood pressure, triglycerides, HDL- and LDL-cholesterol, and fasting and 2-h post-load plasma glucose).

**Results:**

Higher CMR was significantly associated with greater TV-viewing and computer sitting time (*b* [95%CI] = 0.07 [0.04, 0.09] and 0.06 [0.03, 0.09]), and tended to be associated with higher occupational and transport sitting time (0.01 [− 0.01, 0.03] and 0.03 [− 0.00, 0.06]), after adjustment for potential confounders. Furthermore, keeping total sitting time constant, accruing sitting via TV-viewing and computer use was associated with significantly higher CMR (0.05 [0.02, 0.08] and 0.04 [0.01, 0.06]), accruing sitting in an occupational context was associated with significantly lower CMR (− 0.03 [− 0.05, − 0.01]), while no significant association was seen for transport sitting (0.00 [− 0.03, 0.04]). Results varied somewhat between the respective biomarkers; however, higher sitting time in each domain tended to be associated detrimentally with individual biomarkers except for fasting glucose (non-significant associations) and systolic blood pressure (a beneficial association was observed). Overall, associations were stronger for TV-viewing and computer use, and weaker for occupational sitting.

**Conclusions:**

Higher context-specific sitting times tended to be detrimentally associated, albeit modestly, with CMR and several cardiometabolic risk biomarkers. There was some evidence suggesting that the context in which people sit is relevant above and beyond total sitting time. Methodological issues notwithstanding, these findings may assist in identifying priorities for sitting-reduction initiatives, in order to achieve optimal cardiometabolic health benefits.

**Electronic supplementary material:**

The online version of this article (10.1186/s12966-018-0748-3) contains supplementary material, which is available to authorized users.

## Introduction

Sedentary behaviour (sitting) is a prevalent feature of everyday living and is now increasingly being considered as a clinical and public health concern in addition to too little exercise [1]. As opposed to regular engagement in light-intensity activities, a number of metabolic and vascular processes may be significantly dysregulated when sitting for prolonged periods. Prolonged sitting involves less skeletal muscle contractile activity, lower energy expenditure, and low/basal blood flow and vascular shear stress compared with intermittent standing or light activity [2–4], and these processes may adversely impact biomarkers of glucose and lipid metabolism, blood pressure, and adiposity [5–10]. Furthermore, adults can often accumulate 8–10 h or more of sedentary time at work, during transportation and leisure time activities [11, 12], and epidemiological evidence indicates that large volumes of sedentary time, particularly television (TV) viewing time, are associated with elevated cardiometabolic risk profiles in a dose-related manner [13–15]. Accordingly, reducing time spent in sedentary behaviours has the potential to improve cardiometabolic health, and the risk of developing type 2 diabetes or cardiovascular disease.

Arguably, preventive efforts could focus on any and all contexts in which sitting time occurs. However, sitting occurs in multiple settings across the day, and within varying environmental and social contexts — such as watching TV or socialising, working at a computer, driving a car, or sitting at a desk in the workplace. Each of these context-specific settings may have distinct behavioural determinants and health consequences, and thus may require different approaches to intervene [16, 17]. Evidence suggests that not all forms of sitting are equal. Salient concerns that may attenuate or exacerbate the impact of sitting time on cardiometabolic and cognitive outcomes include whether it is accumulated in long or short bouts [18–20], the diurnal profile or metabolic state at the time of the sitting [9, 21], and other social, cognitive or environmental stimuli (e.g., passive vs. active screen time, stress, job control) [22–25]. These considerations, which are often difficult to measure well, could result in sitting time in some contexts being comparatively more or less deleterious, and thus could be a consideration when deciding on how and where to intervene to reduce sedentary time.

Much of the evidence to date examining associations of context-specific sitting time with health outcomes has focused on TV-viewing time [8, 13, 15, 26], which has shown quite consistent adverse associations with disease incidence, mortality and cardiometabolic risk biomarkers [15, 27–30]. By contrast, findings concerning the associations of transportation sitting, reading/gaming, socialising, and/or using a computer with health outcomes have been less consistent [31–33], and the pattern of evidence on occupational sitting remains unclear – particularly in relation to cardiometabolic risk biomarkers [34–36]. While examination of such studies suggests that not all contexts are equal, few studies have concurrently examined sitting in multiple separate contexts in relation to biomarkers of cardiometabolic disease risk, and the unequal contribution of each domain to total sitting time is seldom considered. Evidence on occupational sitting is also particularly sparse in this regard.

In order to inform approaches for the reduction of type 2 diabetes and cardiovascular disease risk, there is the need to better understand the relative importance and contributions of context-specific sitting time. In a large and diverse sample of Australian adults, we examined the associations of sitting time in four contexts (occupational, transport, TV-viewing and leisure-time computer use) with cardiometabolic risk biomarkers.

## Materials and methods

### Participants and procedures

The Australian Diabetes, Obesity and Lifestyle study (AusDiab) is a national longitudinal study, designed originally to examine the prevalence and incidence of diabetes and its precursors in a population-based sample of Australian adults. Details of the data collection methods and response rates have been described previously [37, 38]. Briefly, 11,247 adults participated in the baseline survey in 1999–2000. Follow-up studies were conducted in 2004–05 (AusDiab2) and 2011–12 (AusDiab3). The present study uses data from 4614 participants in AusDiab3, where survey questions on context-specific sitting time were first introduced, and which includes those who attended testing sites for the biomedical examination. In total there were 3973 eligible participants, after excluding those who were pregnant (*n* = 6), clinically diagnosed with diabetes (*n* = 446), or who had a history of cardiovascular disease (angina, heart attack, stroke; *n* = 189). The final sample size was 3429 (1474 men and 1955 women) after excluding those who reported an implausible sitting time (> 18 h on a weekday or weekend day; *n* = 10), or who had missing data on any of the covariates of interest (*n* = 770). The Alfred Hospital Ethics Committee (no. 39/11) approved the study and written informed consent was obtained from all participants.

### Context-specific sitting time

Participants were asked to report sitting time over the past 7 days, separately for weekdays and weekend days, across five contexts (occupational, transport, television viewing, leisure time computer use and “other” sitting). The questions were devised for the AusDiab survey (see Additional file 1; Sitting time questions from AusDiab3). The sum of these five contexts has previously been validated against total sitting time measured by activPAL (*r* = 0.46 [95% CI: 0.40, 0.52]) [39]. Since “other” sitting cannot be meaningfully attributed to a specific context, we did not aim to assess associations between this exposure and the cardiometabolic risk biomarkers. Those who reported not working in “either in a paid position, including self-employment, or as a volunteer” (*n* = 1148) were assigned a zero value for occupational sitting accordingly. Average daily sitting time (h/day) for each of the five contexts [(weekday/5 + weekend/2)/7)] was then calculated. Total sitting time was calculated as the sum of all forms of sitting (including “other”).

### Cardiometabolic risk biomarkers

After an overnight fast (minimum of 10 h) participants attended a local testing centre, where a standard 75 g 2-h oral glucose tolerance test was performed. Fasting serum triglycerides, high density lipoprotein (HDL)-cholesterol, and fasting and 2-h plasma glucose concentrations were measured using the Siemens Advia 2400 (Siemens AG, Munich, Germany) instrument. Trained personnel conducted duplicate waist circumference and resting seated blood pressure measurements. Cardiometabolic risk biomarkers were: waist circumference; systolic and diastolic blood pressure; triglycerides; HDL-cholesterol, low-density lipoprotein (LDL)-cholesterol and LDL/HDL ratio [40]; fasting plasma glucose; and, 2-h plasma glucose. A continuous clustered cardiometabolic risk score (CMR) was computed using waist circumference; blood pressure (average of systolic and diastolic); triglycerides; HDL-cholesterol; and, fasting plasma glucose [41]. After log-normalisation of triglycerides and fasting plasma glucose, all five cardiometabolic variables (average blood pressure was used as an index for systolic and diastolic blood pressure) were standardised, [z = (value-mean)/SD)]. For HDL-cholesterol (protective for cardiometabolic risk), the z-score was inverted. The risk score was then calculated by summing all standardised scores and dividing this sum by five. Higher CMR indicates higher cardiometabolic disease risk.

### Potential confounding variables

Potential confounding variables were determined from interviewer-administered questionnaires. Sociodemographic attributes included age, gender, educational attainment (high school or less, technical/vocational, bachelor’s degree or higher), ethnicity (Australia/New Zealand, other English speaking, other), occupation (professional/managerial, blue collar, white collar/administrative, not currently working) and marital status (married or de facto, not married or de facto). Health-related factors included leisure-time physical activity, alcohol consumption, smoking status, and energy intake). Leisure-time physical activity (h/day), including walking for recreation or transport, other moderate-intensity activity and vigorous-intensity activity, was assessed for the previous week using the Active Australia Survey Questionnaire [42]. Daily energy (kJ/day) and alcohol intake (≤10 g/day, > 10- ≤ 20 g/day, > 20 g/day) were assessed through a self-administered food-frequency questionnaire [43], in which participants reported the frequency of consumption of various food items over the previous 12 months.

### Statistical analyses

A series of multiple linear regression models were conducted to assess the impact of potential confounding variables on the associations of each context-specific sitting variable (occupational, transport, television viewing, and leisure-time computer use) with each cardiometabolic risk variable and a continuous clustered cardiometabolic risk score. Models showed no evidence of collinearity (i.e., variance inflation factor < 2.5), non-linearity, non-normality, or heteroscedasticity as assessed by scatterplots. Biomarkers were examined as continuous measures rather than as binary classifications (e.g., having metabolic syndrome or not), since cardiometabolic risk progressively increases as a function of cardiometabolic risk factors and continuous scores maximise statistical power [44, 45]. Analyses were conducted using Stata-14.1 (StataCorp LP). Statistical significance was set at *P* < 0.05 (two-tailed).

Model A was unadjusted. Model B adjusted for the potential confounders (gender, age, education, ethnicity, occupation, marital status, alcohol intake, and total leisure-time physical activity). Directed acyclic graphs (see Additional file 2: Figures S1-S8) visualise causal assumptions and guided which variables should be included as confounders a priori [46]. The relative contribution of each context to total sitting can produce different results for different contexts, even if all forms of sitting are equally deleterious; therefore, Model C further adjusted for total sitting time. Model C tests whether each context-specific sitting time has an association with cardiometabolic biomarkers that cannot be explained by their contribution to total sitting time, and thus tests whether sitting in a particular context has an association that is different to the remaining contexts combined. In these models, associations that are either in the ‘beneficial’ or ‘detrimental’ direction indicate that sitting accrued in the examined context is respectively less detrimental, or more detrimental, than sitting time accrued outside of the examined context. For simplicity, we have termed these ‘contextual effects’. Interaction terms were used to examine whether the contextual effects examined in model C differed by gender; gender-stratified results are reported. To test whether associations with blood pressure, glucose, and lipids were independent of adiposity (which arguably may be a confounder or a related cause), Model D further adjusted for waist circumference.

Results are expressed in unstandardized effect sizes (*b*) for clinical interpretation, and sometimes also as partially standardised effect sizes (*β*). These *b* and *β* coefficients indicate associations of each additional 1 h/day of context-specific sitting time with biomarkers in units or in standard deviations, respectively. Associations are described as very small (< 0.2 SD), ‘small’ (0.2 SD), ‘medium’ (0.5 SD) and ‘large’ (0.8 SD) for a reasonable dose of sitting time (here, 2 h/day), based on typical interpretations of standardised effect sizes.

## Results

### Descriptive characteristics of the sample

Socio-demographic attributes, health related factors, context-specific sitting time and cardiometabolic risk variables are shown in Table 1. The mean (±SD) age of the sample was 58 ± 10 years. Of the participants, 57% were women, 65% reported meeting the minimum leisure-time physical activity recommendations, 68% were overweight or obese, and approximately one third (33.5%) were not working while the remainder worked in professional/managerial occupations (34.4%) white collar/administrative roles (20.2%) or blue-collar jobs (11.9%). Total self-reported sitting time averaged 6.8 ± 2.8 h/day, with similar amounts of sitting time reportedly spent in the occupational (1.8 ± 2.3 h/day), TV-viewing (1.8 ± 1.3 h/day) and “other” (1.7 ± 1.3) contexts, and less sitting time in the transportation (0.8 ± 0.8 h/day) and computer (0.6 ± 0.9 h/day) contexts. Compared with eligible participants included in the analytic sample, eligible participants who were excluded due to missing data were significantly older, less physically active, sat less in occupational settings, watched more television, had poorer overall cardiometabolic risk profiles, and differed in relation to gender, education, occupation and marital status distributions (see Additional file 3: Table S1). Sociodemographic and cardiometabolic risk differences between the analytic and excluded samples were largely attenuated after adjustment for age.

**Table 1:** Sociodemographic attributes, health-related and behavioural factors, and cardiometabolic risk variables of the sample according to gender

	Total sample (*n* = 3429)	Men (*n* = 1474)	Women (*n* = 1955)
Socio-demographic attributes			
Age (y)	58 ± 10	58 ± 10	58 ± 10
Parental history of diabetes, *n* (%)	968 (28.2)	385 (26.1)	583 (29.8)
Education, *n* (%)			
High school or less	1082 (31.6)	344 (23.3)	738 (37.7)
Technical/vocational	1488 (43.4)	717 (48.6)	771 (39.4)
Bachelor’s degree or higher	859 (25.1)	413 (28.0)	446 (22.8)
Ethnicity, *n* (%)			
Australia/New Zealand	2725 (79.5)	1151 (78.1)	1574 (80.5)
Other English speaking	411 (12.0)	179 (12.1)	232 (11.9)
Other	293 (8.5)	144 (9.8)	149 (7.6)
Occupation, *n* (%)			
Professional/managerial	1180 (34.4)	603 (40.9)	577 (29.5)
Blue collar	408 (11.9)	319 (21.6)	89 (4.6)
White collar/administrative	693 (20.2)	133 (9.0)	560 (28.6)
Not currently working	1148 (33.5)	419 (28.4)	729 (37.3)
Marital status, *n* (%)			
Married or de facto	2763 (80.6)	1273 (86.4)	1490 (76.2)
Not married or de facto	666 (19.4)	201 (13.6)	465 (23.8)
Health-related factors			
Total physical activity time (h/day)	0.9 ± 0.9	1.0 ± 0.9	0.8 ± 0.8
Energy intake (kJ/day)	7164 ± 2794	8349 ± 3042	6270 ± 2204
Alcohol consumption, *n* (%)			
≤ 1 standard drinks/day	1898 (55.4)	621 (42.1)	1277 (65.3)
> 1–2 standard drinks/day	589 (17.2)	261 (17.7)	328 (16.8)
> 2 standard drinks/day	942 (27.5)	592 (40.2)	350 (17.9)
Smoking status, *n* (%)			
Current or ex-smoker	1337 (39.9)	656 (45.5)	681 (35.6)
Non-smoker	2017 (60.1)	785 (54.5)	1232 (64.4)
Sitting time spent in specific contexts (h/day)			
Occupational	1.8 ± 2.3	2.3 ± 2.5	1.4 ± 2.0
Transportation	0.8 ± 0.8	0.9 ± 0.9	0.8 ± 0.7
TV viewing	1.8 ± 1.3	1.9 ± 1.2	1.8 ± 1.3
Computer use	0.6 ± 0.9	0.7 ± 0.9	0.6 ± 1.0
Other	1.7 ± 1.3	1.5 ± 1.2	1.7 ± 1.3
Total	6.8 ± 2.8	7.4 ± 2.9	6.4 ± 2.7
Cardiometabolic risk variables			
2-h plasma glucose (mmol/L)	5.8 ± 2.0	5.9 ± 2.1	5.7 ± 1.9
Fasting plasma glucose (mmol/L)	5.3 ± 0.7	5.5 ± 0.7	5.2 ± 0.6
Systolic blood pressure (mmHg)	128 ± 18	132 ± 17	125 ± 18
Diastolic blood pressure (mmHg)	73 ± 11	75 ± 11	72 ± 11
Triglycerides (mmol/L)	1.3 ± 0.8	1.5 ± 1.0	1.2 ± 0.7
HDL-cholesterol (mmol/L)	1.6 ± 0.4	1.3 ± 0.3	1.7 ± 0.4
LDL/HDL ratio (mmol/L)	2.1 ± 0.8	2.4 ± 0.8	1.9 ± 0.7
BMI (kg/m^2^)	27.6 ± 5.1	27.9 ± 4.3	27.4 ± 5.6
Waist circumference (cm)	93.7 ± 13.9	100.1 ± 11.9	88.9 ± 13.3
Clustered cardiometabolic risk (z)	0.0 ± 0.6	0.3 ± 0.6	-0.3 ± 0.6

Data are means ± SD, or n (%), corrected for complex survey design

### Associations of context-specific sitting time with cardiometabolic risk biomarkers

The associations of context-specific sitting time with cardiometabolic risk biomarkers, unadjusted and adjusted for potential confounders, are shown in Table 2. Unadjusted models showed statistically significant associations of context-specific sitting time and cardiometabolic biomarker outcomes (either beneficial or adverse in direction). Associations were of ‘very small’ magnitude, and occasionally of a ‘small’ magnitude for sitting during TV viewing and computer use.

**Table 2:** Associations of context-specific sitting time (h/day) with biomarkers of cardiometabolic risk

		Occupational (h/day)	Transport (h/day)	TV viewing (h/day)
Cardiometabolic Outcome	Model	*b* (95% CI)	*b* (95% CI)	*b* (95% CI)
2-h plasma glucose (mmol/L)	A	-0.06 (-0.10, -0.03) ***	0.07 (-0.02, 0.16)	0.17 (0.11, 0.23)***
	B	0.04 (-0.00, 0.08)	0.12 (0.04, 0.20)**	0.06 (0.00, 0.12)
Fasting plasma glucose (mmol/L)	A	0.00 (-0.01, 0.01)	0.01 (-0.03, 0.04)	0.04 (0.02, 0.06)**
	B	0.01 (-0.00, 0.02)	0.01 (-0.02, 0.04)	0.01 (-0.01, 0.03)
Systolic blood pressure (mmHg)	A	-1.24 (-1.45, -1.02)***	-0.78 (-1.45, -0.11)*	1.82 (1.20, 2.43)***
	B	-0.45 (-0.70, -0.19)**	-0.43 (-0.99, 0.14)	0.45 (-0.13, 1.03)
Diastolic blood pressure (mmHg)	A	0.10 (-0.07, 0.27)	0.21 (-0.26, 0.68)	0.58 (0.25, 0.91)**
	B	-0.15 (-0.37, 0.08)	0.06 (-0.40, 0.52)	0.51 (0.16, 0.85)**
Triglycerides (mmol/L)	A	0.02 (0.00, 0.03)**	0.03 (-0.01, 0.06)	0.05 (0.03, 0.08)**
	B	0.02 (0.00, 0.03)*	0.02 (-0.01, 0.05)	0.04 (0.01, 0.06)**
HDL-cholesterol (mmol/L)	A	-0.02 (-0.03, -0.02)***	-0.03 (-0.05, -0.01)**	-0.03 (-0.04, -0.02)***
	B	-0.01 (-0.01, 0.00)	-0.01 (-0.03, 0.00)	-0.02 (-0.03, -0.01)**
LDL/HDL ratio (mmol/L)	A	0.04 (0.03, 0.06)***	0.06 (0.03, 0.09)**	0.03 (0.01, 0.05)**
	B	0.02 (-0.00, 0.03)	0.02 (-0.00, 0.05)	0.03 (0.01, 0.06)**
BMI (kg/m^2^)	A	0.05 (-0.03, 0.13)	0.20 (0.01, 0.38)*	0.35 (0.21, 0.49)***
	B	0.11 (-0.01, 0.22)	0.22 (0.04, 0.41)*	0.27 (0.12, 0.42)**
Waist circumference (cm)	A	0.29 (0.12, 0.47)**	0.95 (0.42, 1.49)**	1.41 (1.11, 1.72)***
	B	0.23 (-0.01, 0.47)	0.66 (0.17, 1.16)**	0.70 (0.41, 1.00)***
Clustered cardiometabolic risk (z)	A	0.01 (-0.00, 0.03)	0.05 (0.01, 0.09)*	0.13 (0.10, 0.15)***
	B	0.01 (-0.01, 0.03)	0.03 (-0.00, 0.06)	0.07 (0.04, 0.09)***

Model A: unadjusted model

Model B: adjusted for age, gender, education, ethnicity, occupation, marital status, alcohol intake and leisure-time physical activity

*p < 0.05; **p < 0.01; ***p < 0.0001

Confounding likely partly explained some of the unadjusted findings. After statistical adjustment for potential confounders, most of these associations seen in unadjusted models were attenuated in magnitude and many were no longer statistically significant (Table 2). In the adjusted models, all the cardiometabolic biomarkers except for blood pressure became worse with higher amounts of context-specific sitting time. Effect sizes were small in magnitude in the case of the associations between sitting for computer use and BMI, but were otherwise very small. The CMR score, which is comprised of a number of these separate cardiometabolic biomarkers (including blood pressure), was significantly higher with higher sitting in the computer use and TV-viewing contexts. Transport and computer use sitting were associated with higher 2-h plasma glucose by a very small to small degree. All the lipid outcomes and adiposity indicators were significantly worse with higher levels of sitting for TV viewing and computer use. Further, triglycerides were significantly higher with increased occupational sitting and higher BMI and waist circumference were seen with higher sitting for transport. Unlike the other biomarkers, systolic and diastolic blood pressure tended to either decrease or increase to a very small degree with higher context-specific sitting time. Systolic blood pressure tended to increase with sitting for TV or computer use, but, unlike the other cardiometabolic biomarkers, significantly decreased with higher levels of occupational sitting and tended to decrease with sitting for transport. Diastolic blood pressure significantly increased with higher TV viewing, tended to increase with higher sitting for computer use and transport, but tended to decrease slightly with higher occupational sitting.

### Differences between contexts

**Fig. 1:**
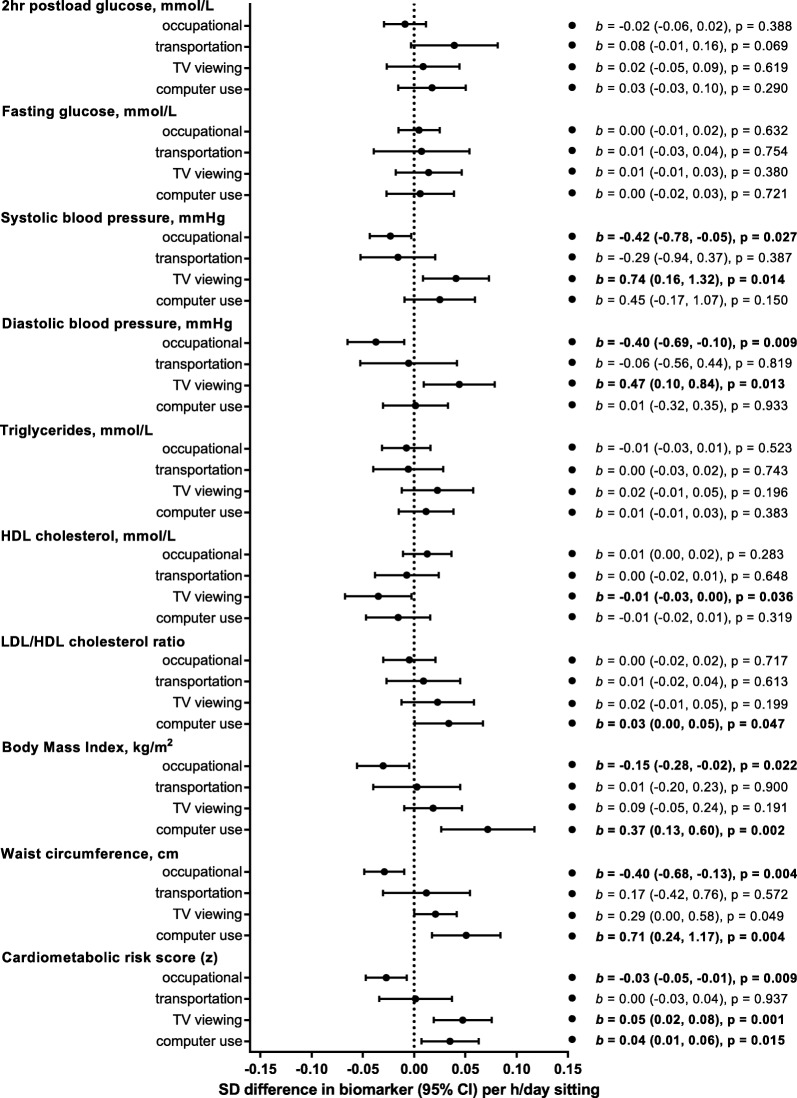
Associations (95% CI) of context-specific sitting time (h/day) with cardiometabolic risk biomarkers, adjusted for total sitting time and potential confounders. The standardised (*β*) effect sizes are plotted and the unstandardised effect sizes (*b*) are shown in text

The findings above were suggestive of some differences between contexts and their associations with cardiometabolic biomarkers; results of the formal tests (Model C) are shown in Fig. 1. Significant contextual effects (i.e., significant associations of context-specific sitting, independent of total sitting time), were seen in relation to CMR (occupational, TV-viewing, computer use), BMI (occupational, computer use), waist circumference (occupational, TV-viewing, computer use), HDL-cholesterol (TV-viewing), and both systolic and diastolic blood pressure (occupational, TV-viewing, computer use). All the effect sizes (significant and non-significant) were of a very small magnitude. The direction of the associations consistently showed sitting in the TV-viewing and computer use contexts either had (or tended to have) associations with biomarkers that were always equally or more deleterious than sitting outside these contexts. Conversely, occupational sitting was (or tended to be) less detrimentally associated with biomarkers than was sitting outside of this context. Sitting for transportation did not appear to have consistent directional associations (beneficial or adverse), all of which were non-significant.

### Relevance of adiposity

The associations of context-specific sitting time with glucose, lipids and blood pressure, having adjusted for total sitting time, were mostly unchanged by further adjustment for waist circumference, with minimal changes to effect sizes (Additional file 4: Figure S9). The tendency was mostly for effects to move slightly closer towards the null upon adjustment, but only slightly so. The only associations to change in statistical significance were those for TV-viewing with HDL-cholesterol (*p* = 0.071), which occurred with no change in effect size, and for occupational sitting with systolic blood pressure (*p* = 0.097), which was attenuated slightly in magnitude (− 0.42 vs. − 0.31 mmHg per h/day).

### Differences by gender

Some of the contextual effects varied significantly by gender (Additional file 3: Table S2). All the gender differences were of a very small to sometimes small magnitude. The tendency for occupational sitting to be less harmful relative to other forms of sitting was more pronounced in women than in men for CMR, systolic blood pressure, BMI and waist circumference to a very small degree, and plausibly triglycerides to a small degree (based on confidence intervals). Similarly, the tendency for sitting as part of TV-viewing to be more harmful relative to other forms of sitting was also more pronounced in women than in men for CMR, triglycerides, and 2-h plasma glucose to a small degree, while more favourable associations were observed for women (detrimental for men) with computer use and 2-h plasma glucose only to a small degree. Associations of transport sitting with outcomes did not vary significantly, or to more than a small degree, by gender.

## Discussion

In this large sample of Australian adults, self-reported time spent sitting in specific contexts (occupational, transport, TV-viewing and leisure-time computer use) showed small or very small, statistically significant associations (usually detrimental) with a range of cardiometabolic biomarkers. Except for blood pressure, greater volumes of sitting time tended to relate to poorer cardiometabolic biomarker profiles. These results varied depending on both the biomarker and the context, with effect sizes typically being largest for the adiposity markers and CMR, and for sitting in leisure contexts (TV-viewing and computer use). Only very small and non-significant contextual effects were seen for sitting for transportation. Associations with biomarkers tended to be more detrimental for sitting time in the TV-viewing and computer use contexts than for sitting time outside these contexts, while the converse was seen for sitting in the occupational context, with some of these differences reaching statistical significance. These findings provide some evidence that the context of sitting itself may be important, not just the amount of sitting time, potentially due to methodological and/or biological phenomena.

Our findings are consistent with prior research using self-report measures [15, 27–30, 47–49] which have reported stronger deleterious associations for TV-viewing with a number of cardiometabolic risk biomarkers than are sometimes seen with other sitting time measures. Extending on previous research, we accounted for total sitting time and in doing so, we formally tested whether sitting as part of TV-viewing had different associations with cardiometabolic risk than sitting time outside these contexts — observing this to sometimes be the case. Computer use has previously been shown to be inconsistently associated with cardiometabolic risk biomarkers [50–55]. However, computer use and TV-viewing time are often combined into single ‘leisure-time’ or ‘screen-time’ metrics [56, 57]. Our results partly supported considering TV-viewing and computer use collectively in relation to biomarkers, as the results were similar (but not identical) for sitting in these two leisure contexts, whereas the same could not be said of sitting in the occupational and transport contexts. In this context, and with the wider and more concurrent use of online streaming and mobile/tablet devices in recent years, the distinguishing line between TV-viewing and computer use is now being increasingly blurred. With this in mind, it may be sufficient for future surveys to include a “screen time” measure that would encompass all such activities that are done while seated, at least where cardiometabolic risk is concerned.

Few prior studies have concurrently included a direct, continuous measure of occupational sitting time when assessing associations of context-specific sitting with health outcomes, often instead relying on categorical measures of occupational physical activity, or indirect measures, classifying the exposure based on the estimated main activity of a person’s occupation (e.g., mostly sitting, mostly standing) [34]. Findings have been limited and/or mixed regarding the relationships between occupational sitting per se and indicators of cardiometabolic health [34, 35, 58]. Adding to these mixed findings, we mostly saw weak non-significant associations, except in relation to triglycerides (very small detrimental association) and systolic blood pressure (very small, beneficial association). Further, we observed that sitting time in the occupational context tended to be less adversely associated with several biomarkers than did sitting outside of this context. Broadly, our results support the extant literature concerning the relevance of different domains and concerning stronger relationships with some cardiometabolic risk biomarkers for women compared to men (11, 20–22). However, previous studies have largely been limited to TV-viewing exposures, and evidence remains unclear in relation to other sitting contexts such as occupational sitting (17).

Our study does not have the detailed data that would be required to establish why the contextual variations in cardiometabolic biomarkers occurred. Although not definitive at present, some observational research has suggested that TV-viewing — by comparison with other sedentary behaviours such as reading and computer use — may be more strongly associated with unhealthy dietary patterns or behaviours such as snacking [22, 23, 59], which could lead to different cardiometabolic risk sequelae. Furthermore, TV-viewing often involves more prolonged uninterrupted sitting and is more passive in nature. It also tends to occur after a large evening meal (particularly in Western societies) and at a time when liver/peripheral insulin sensitivity and lipid trafficking are suboptimal in part due to circadian chronobiology [60, 61]. Our study included only total dietary intake measures, not time-based measures, and similarly measures of total volume of sitting in each context, not of when and for how long the sitting occurred in prolonged bouts. Such data would be informative in future research, although inevitably more complex to interpret.

In addition to the potential mechanisms above, some methodological limitations in our study, and in much of the extant literature that could produce apparent differences between contexts, should be considered. One possible explanation for the relative benefit of occupational sitting over other forms of sitting, and for the beneficial association with systolic blood pressure, is the ‘healthy worker’ effect [62]. Another possibility is confounding from unmeasured considerations pertinent to employment status (e.g., some facets of socioeconomic position, job control, stress and/or shift-related work) [63]. These same issues could be relevant in terms of gender differences in contextual effects, as it is plausible that a range of employment characteristics may vary by gender. Indeed, a lower proportion of women were exposed to full time work in the present study sample. Differential measurement error is also a possibility as our study, like most, relied on self-report data. It is possible that some sitting contexts (e.g., TV viewing) correlate more strongly with biomarkers because they are more accurately measured than others, being either easier to recall accurately, or better correlated with the true underlying average due to them being more habitual and thus less variable over time [39, 56]. This issue may also apply to gender differences, with differential validity and/or reliability sometimes seen between men and women [64]. These limitations could possibly be reduced in the future with long-term monitoring via ecological momentary assessment [65] — a method that is increasingly feasible via smartphone technologies. Further measurement improvements include proximity tagging, which has successfully measured time in the office, and might prove suitable for measuring contexts attributable to particular locations [66–68]. Electronic monitoring of media content is another objective option; however, this is at present expensive and difficult to attribute to a specific individual while sitting. All these options allow for the collection of date-time-stamped data, which may further help elucidate some of the possible mechanisms concerning timing, especially if date-time-stamped data on dietary intake is collected concurrently.

Strengths of this study include the large and diverse sample, the simultaneous investigation of four continuous context-specific sitting measures, and the use of a range of continuous biomarker outcomes collected in a fasting state. Another strength was that the sample size was sufficient to either detect a significant association or rule out all but small effects as unlikely (based on the confidence intervals). In addition to the previously-mentioned limitations, this was a secondary analysis, and the cross-sectional design does not provide insights into the temporality of the associations. Furthermore, the sample was not population representative and there may be some selection biases. In particular, the analysis focused on a healthy sample within the third wave of a cohort that had some loss to follow up [69] and there were some differences between participants included and those excluded due to missing data. As such, young adults were not represented within the sample and results should not be automatically be generalised to this population.

## Conclusions

In this sample of Australian adults, higher volumes of context-specific sitting time were associated detrimentally to a small or very small degree with several biomarkers of cardiometabolic risk — particularly adiposity, some lipids and CMR — with relationships dependent upon the biomarker examined, and the context in which the sitting time was reported. Context was important ‘above and beyond’ the total amount of sitting time, with TV-viewing and computer use contexts tending to be more detrimental than sitting time outside of these contexts, and occupational sitting tending to be less deleterious than non-occupational sitting. Overall, findings lend further support to the notion that not all forms of sitting are equally related to cardiometabolic risk. This may assist in identifying priorities for sitting-reduction initiatives, in order to achieve optimal or additional cardiometabolic health benefits. Building this evidence-base may also assist in the formulation of preventive initiatives required to address excessive sitting time, as part of an integrated approach to reducing the impact of physical inactivity at the population level.

## Additional files

Additional file 1: Sitting time questions from AusDiab3. (PDF 112 kb)

Additional file 2: Directed acyclic graphs of causal assumptions and confounding. (PDF 1728 kb)

Additional file 3: **Table S1.** Characteristics of eligible participants who were included or excluded due to missing data. **Table S2.** Associations of context-specific sitting time (h/day) with biomarkers of cardiometabolic risk: stratified by gender. (DOCX 28 kb)

Additional file 4: **Figure S9.** Associations (95% CI) of context-specific sitting time (h/day) with cardiometabolic risk biomarkers, adjusted for potential confounders, total sitting time and further for waist circumference. Associations are plotted standardised (*β*) and shown in text unstandardised (*b*). (PDF 66 kb)
