# Supplementary material for: Associations of context-specific sitting time with markers of cardiometabolic risk in Australian adults
Source: Int J Behav Nutr Phys Act. 2018 Nov 20;15:114. doi: 10.1186/s12966-018-0748-3 (PMC6245709; doi:10.1186/s12966-018-0748-3)
Supplement: Supplementary file 2 — Directed acyclic graphs of causal assumptions and confounding. (PDF 1728 kb) [file 12966_2018_748_MOESM2_ESM.pdf]

LTPA, Leisure-time physical activity
CMB-R, Cardiometabolic risk

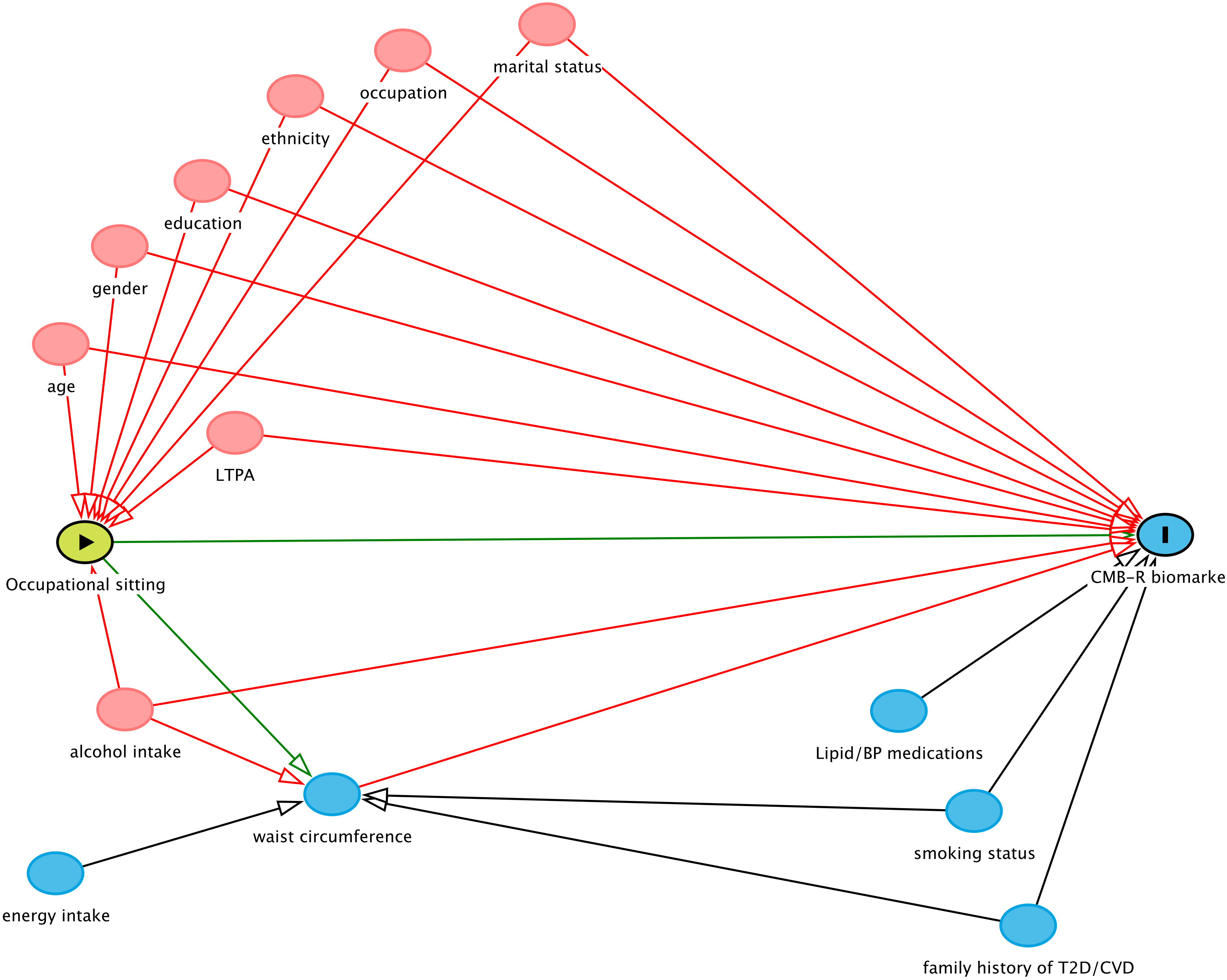

Directed Acyclic Graphs, Figure S1

LTPA, Leisure-time physical activity
CMB-R, Cardiometabolic risk

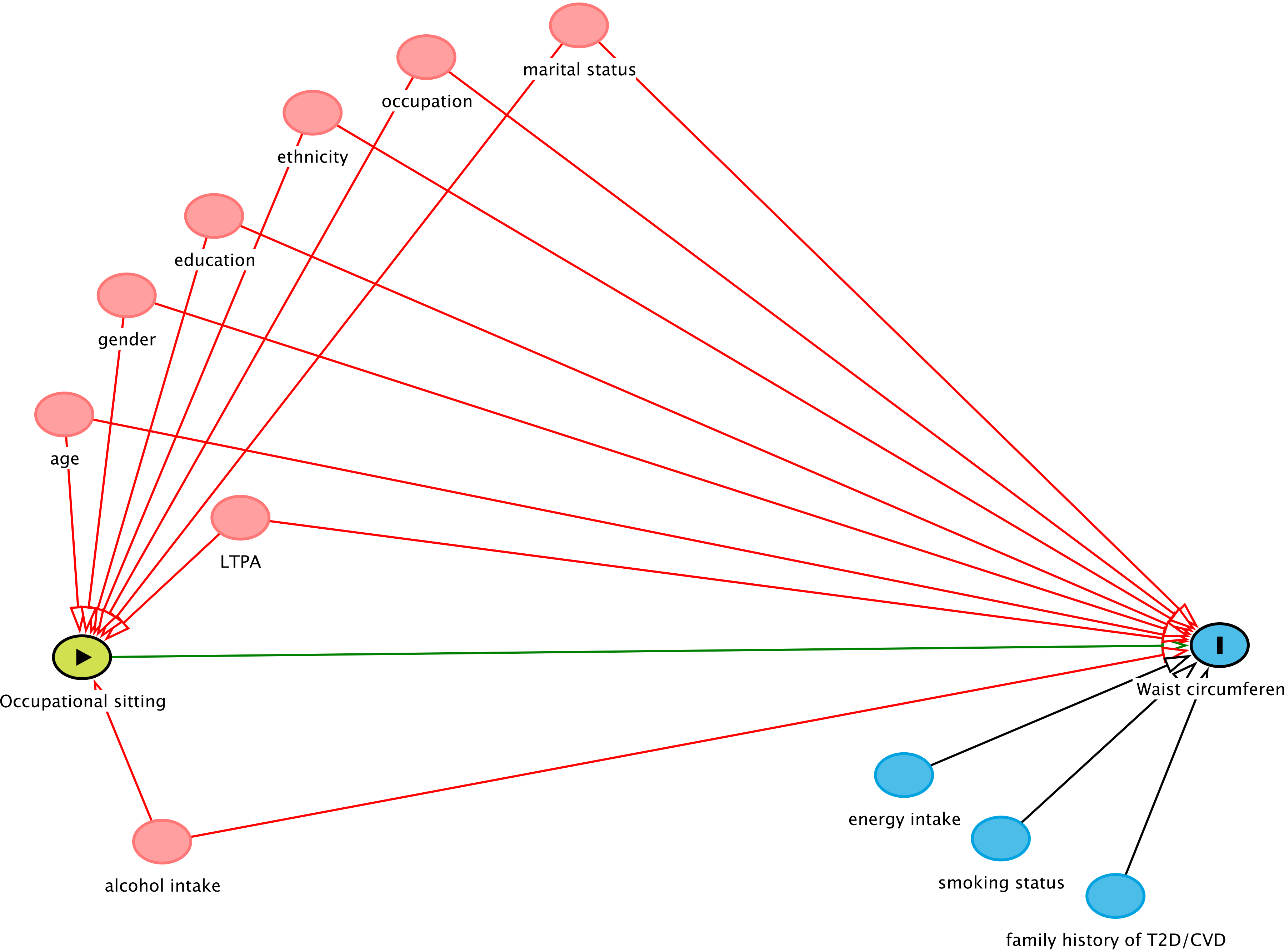

Directed Acyclic Graphs, Figure S2

LTPA, Leisure-time physical activity
CMB-R, Cardiometabolic risk

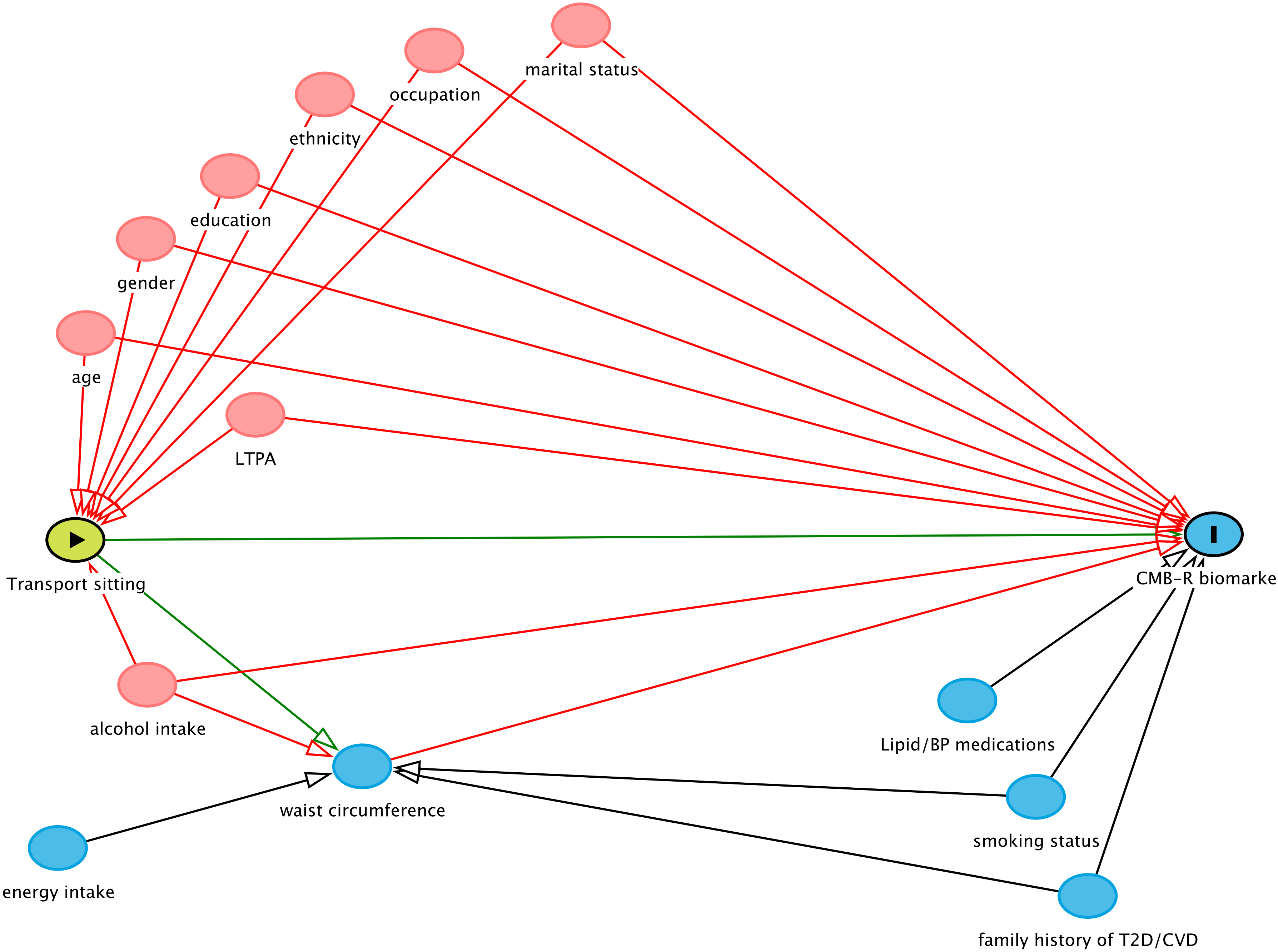

Directed Acyclic Graphs, Figure S3

LTPA, Leisure-time physical activity
CMB-R, Cardiometabolic risk

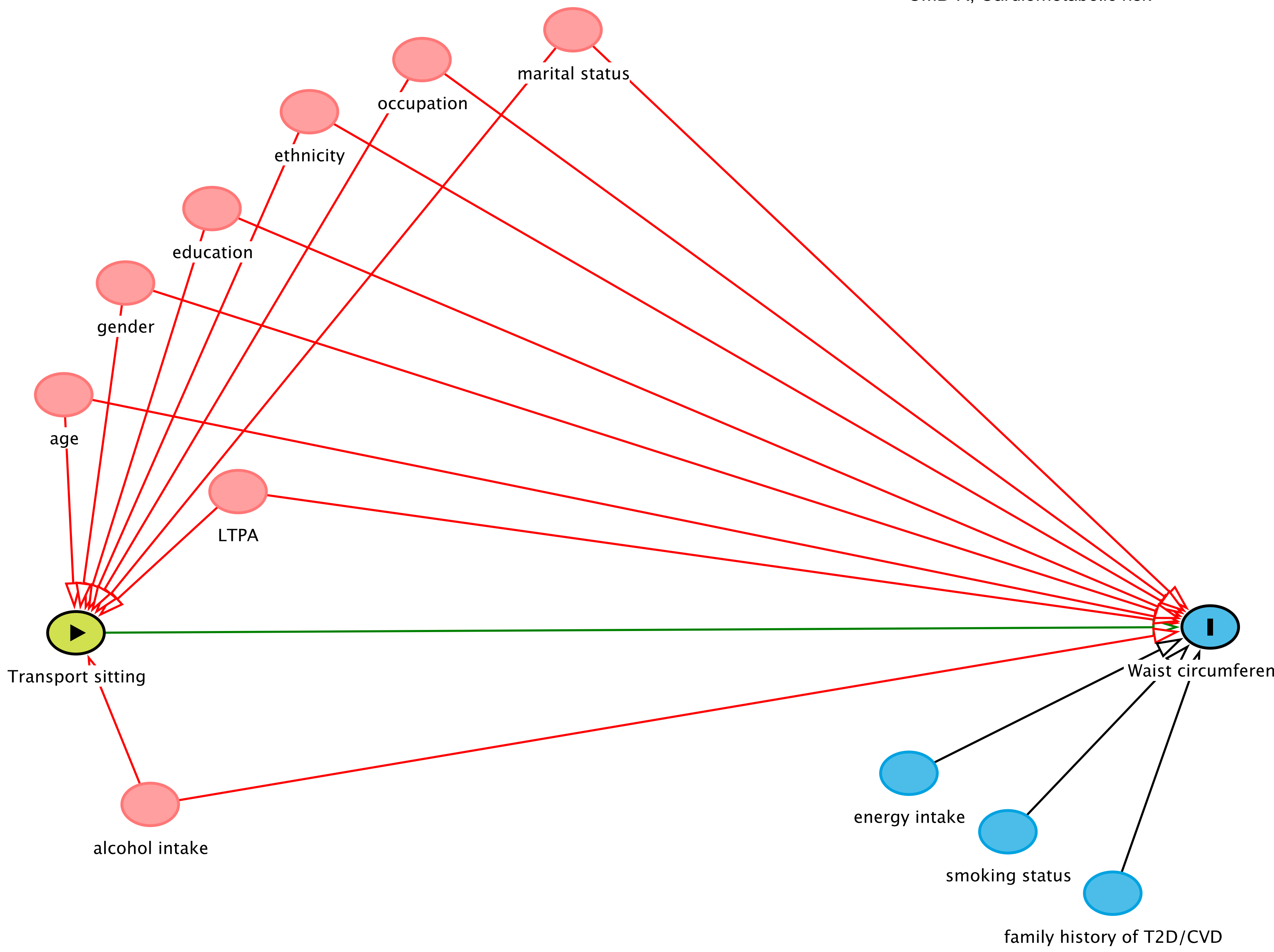

Directed Acyclic Graphs, Figure S4

LTPA, Leisure-time physical activity
CMB-R, Cardiometabolic risk

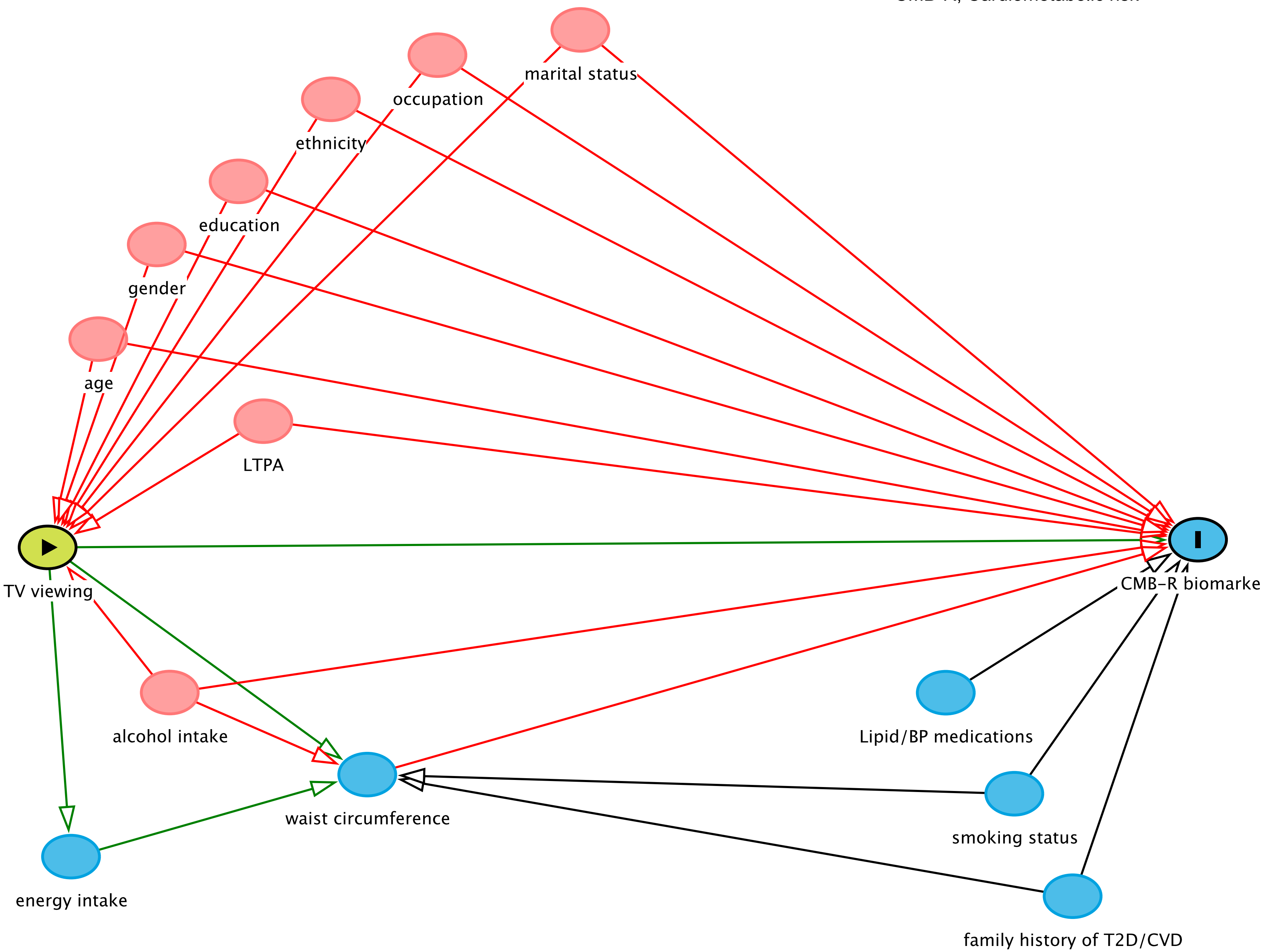

Directed Acyclic Graphs, Figure S5

LTPA, Leisure-time physical activity
CMB-R, Cardiometabolic risk

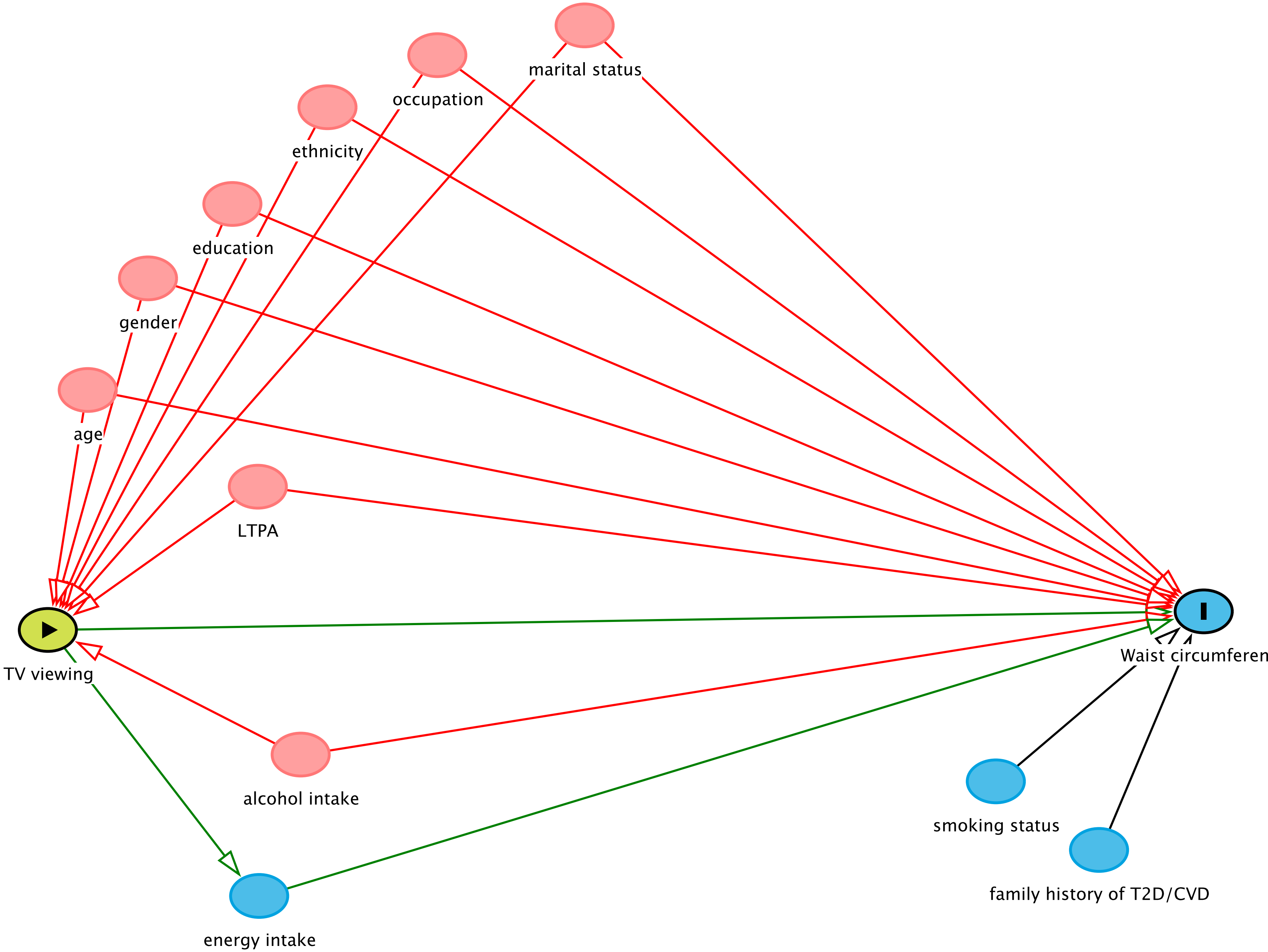

Directed Acyclic Graphs, Figure S6

LTPA, Leisure-time physical activity
CMB-R, Cardiometabolic risk

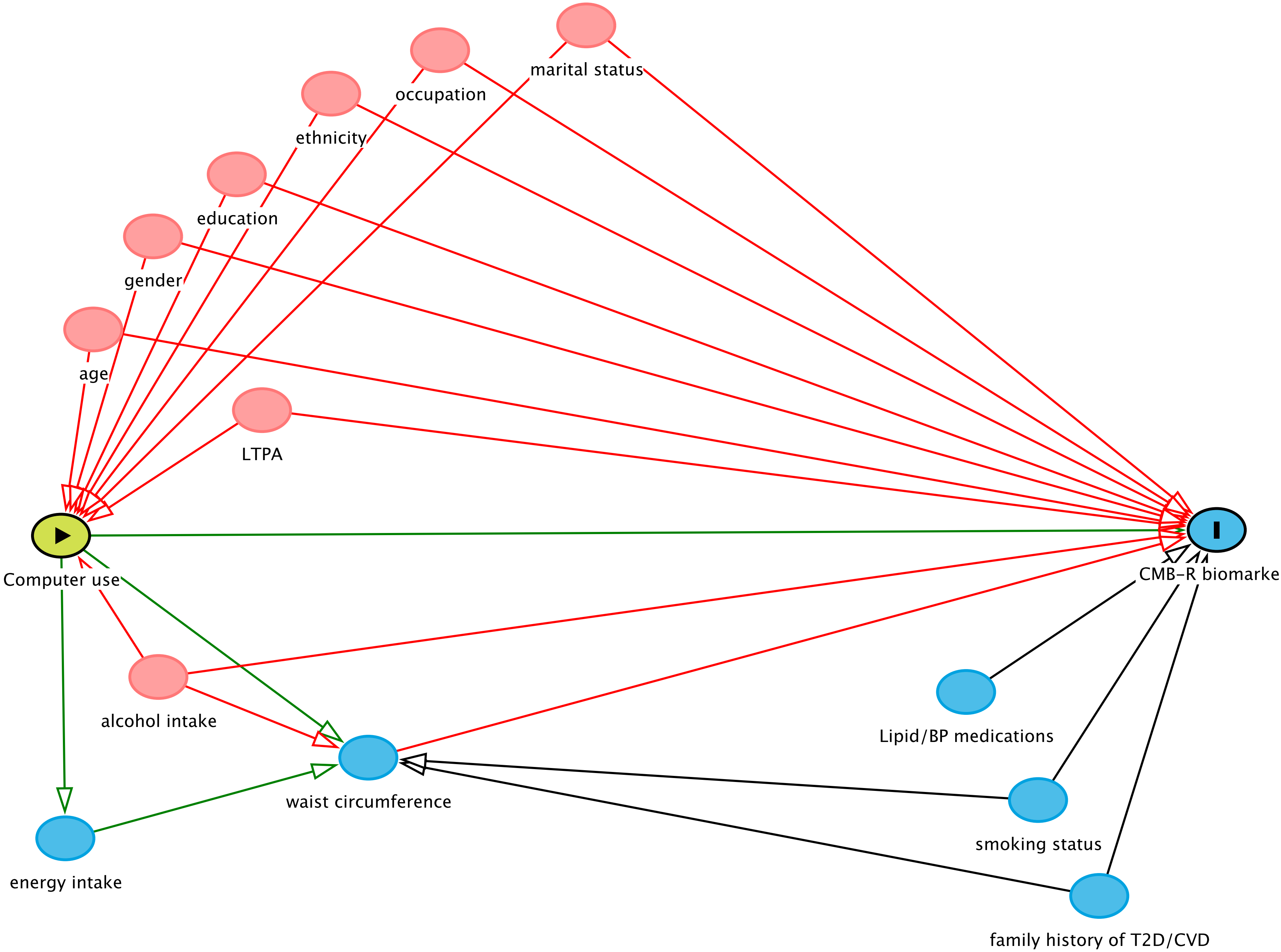

Directed Acyclic Graphs, Figure S7

LTPA, Leisure-time physical activity
CMB-R, Cardiometabolic risk

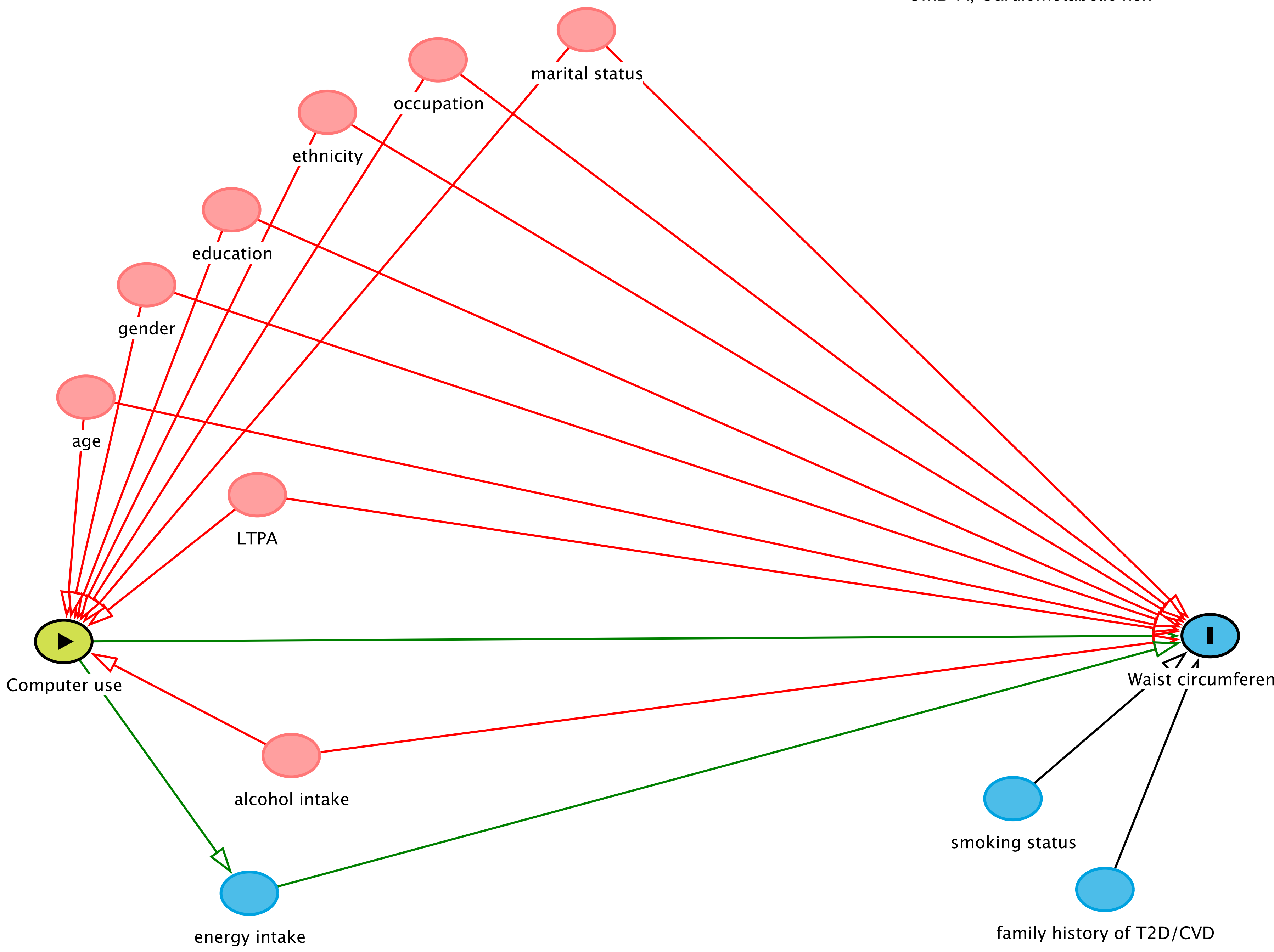

Directed Acyclic Graphs, Figure S8
