# Supplementary material for: Associations of context-specific sitting time with markers of cardiometabolic risk in Australian adults
Source: Int J Behav Nutr Phys Act. 2018 Nov 20;15:114. doi: 10.1186/s12966-018-0748-3 (PMC6245709; doi:10.1186/s12966-018-0748-3)
Supplement: Supplementary file 3 — Table S1. Characteristics of eligible participants who were included or excluded due to missing data. Table S2. Associations of context-specific sitting time (h/day) with biomarkers of cardiometabolic risk: stratified by gender. (DOCX 28 kb) [file 12966_2018_748_MOESM3_ESM.docx]

**Table S1 – Characteristics of** **eligible participants who were included or excluded due to missing data.**

|  | **Analytic sample (n=3,429)** | **Excluded sample (n=779)** | ***p*^a^** | ***p*^b^** |  |
| --- | --- | --- | --- | --- | --- |
| **Socio-demographic attributes** |  |  |  |  |  |
| Gender (male), n (%) | 1,474 (43.0) | 376 (48.3) | 0.012 | 0.108 |  |
| Age (y) | 58 ± 10 | 71 ± 13 | < .001 | - |  |
| Parental history of diabetes, n (%) | 968 (28.2) | 179 (26.5) | 0.443 | 0.182 |  |
| Education, n (%) |  |  | < .001 | 0.428 |  |
| *High school or less* | 1,082 (31.6) | 297 (39.5) |  |  |  |
| *Technical/vocational* | 1,488 (43.4) | 335 (44.6) |  |  |  |
| *Bachelor’s degree or higher* | 859 (25.1) | 119 (15.8) |  |  |  |
| Ethnicity, n (%) |  |  | 0.049 | 0.299 |  |
| *Australia/New Zealand* | 2,725 (79.5) | 592 (76.0) |  |  |  |
| *Other English speaking* | 411 (12.0) | 90 (11.6) |  |  |  |
| *Other* | 293 (8.5) | 97 (12.5) |  |  |  |
| Occupation, n (%) |  |  | < .001 | 0.105 |  |
| *Professional/managerial* | 1,180 (34.4) | 37 (10.0) |  |  |  |
| *Blue collar* | 408 (11.9) | 25 (6.8) |  |  |  |
| *White collar/administrative* | 693 (20.2) | 30 (8.1) |  |  |  |
| *Not currently working* | 1,148 (33.5) | 277 (75.1) |  |  |  |
| Marital status, n (%) |  |  | < .001 | 0.229 |  |
| *Married or de facto* | 2,763 (80.6) | 464 (69.6) |  |  |  |
| *Not married or de facto* | 666 (19.4) | 203 (30.4) |  |  |  |
|  |  |  |  |  |  |
| **Health-related factors** |  |  |  |  |  |
| Total physical activity time (h/day) | 0.9 ± 0.9 | 0.7 ± 0.8 | 0.001 | 0.038 |  |
| Energy intake (kJ/day) | 7,164 ± 2,794 | 7,022 ± 2,979 | 0.365 | 0.610 |  |
| Alcohol consumption, n (%) |  |  | 0.145 | 0.390 |  |
| *≤1 standard drinks/day* | 1,898 (55.4) | 212 (58.7) |  |  |  |
| *>1-2 standard drinks/day* | 589 (17.2) | 62 (17.2) |  |  |  |
| *>2 standard drinks/day* | 942 (27.5) | 87 (24.1) |  |  |  |
| Smoking status, n (%) |  |  | 0.384 | 0.725 |  |
| *Current or ex-smoker* | 1,337 (39.9) | 270 (41.9) |  |  |  |
| *Non-smoker* | 2,017 (60.1) | 375 (58.1) |  |  |  |
|  |  |  |  |  |  |
| **Sitting time spent in specific contexts (h/day)** |  |  |  |  |  |
| Occupational | 1.8 ± 2.3 | 1.0 ± 1.9 | < .001 | < .001 |  |
| Transportation | 0.8 ± 0.8 | 0.7 ± 0.8 | 0.001 | 0.670 |  |
| TV viewing | 1.8 ± 1.3 | 2.1 ± 1.4 | < .001 | 0.192 |  |
| Computer use | 0.6 ± 0.9 | 0.6 ± 1.1 | 0.144 | 0.278 |  |
| Other | 1.7 ± 1.3 | 2.2 ± 1.7 | < .001 | 0.277 |  |
| Total | 6.8 ± 2.8 | 6.6 ± 3.3 | 0.106 | < .001 |  |
|  |  |  |  |  |  |
| **Cardiometabolic risk variables** |  |  |  |  |  |
| 2-h plasma glucose (mmol/L) | 5.8 ± 2.0 | 6.2 ± 2.1 | < .001 | 0.187 |  |
| Fasting plasma glucose (mmol/L) | 5.3 ± 0.7 | 5.6 ± 1.5 | < .001 | 0.004 |  |
| Systolic blood pressure (mmHg) | 128 ± 18 | 137 ± 23 | < .001 | 0.566 |  |
| Diastolic blood pressure (mmHg) | 73 ± 11 | 71 ± 11 | < .001 | 0.278 |  |
| Triglycerides (mmol/L) | 1.3 ± 0.8 | 1.3 ± 0.7 | 0.695 | 0.654 |  |
| HDL-cholesterol (mmol/L) | 1.6 ± 0.4 | 1.5 ± 0.4 | 0.016 | 0.001 |  |
| LDL/HDL ratio (mmol/L) | 2.1 ± 0.8 | 2.0 ± 0.8 | < .001 | 0.455 |  |
| BMI (kg/m^2^) | 27.6 ± 5.1 | 27.3 ± 4.8 | 0.028 | 0.969 |  |
| Waist circumference (cm) | 93.7 ± 13.9 | 95.3 ± 13.7 | 0.008 | 0.519 |  |
| Clustered cardiometabolic risk (z) | 0.0 ± 0.6 | 0.1 ± 0.7 | < .001 | 0.040 |  |

Data are means ± SD, or n (%), corrected for complex survey design.

^a^ *p* for difference between included and excluded participants.

^b^ age adjusted *p* for difference between included and excluded participants.

**Table S2 – Associations of context-specific sitting time (h/day) with biomarkers of cardiometabolic risk: stratified by gender**

|  |  |  | **Occupational** |  | **Transport** |  | **TV viewing** |  | **Computer** |
| --- | --- | --- | --- | --- | --- | --- | --- | --- | --- |
| **Cardiometabolic Outcome** | **Gender** |  | ***b* (95% CI)** |  | ***b* (95% CI)** |  | ***b* (95% CI)** |  | ***b* (95% CI)** |
| 2-h plasma glucose (mmol/L) |  | *p = .965* | | *p = .874* | | ***p = .040*** | | ***p = .019*** | |
|  | Men |  | 0.00 (-0.06, 0.06) |  | 0.07 (-0.06, 0.20) |  | -0.08 (-0.19, 0.02) |  | 0.09 (-0.02, 0.20) |
|  | Women |  | 0.00 (-0.05, 0.06) |  | 0.06 (-0.04, 0.17) |  | 0.07 (0.00, 0.14) |  | -0.06 (-0.12, 0.01) |
| Fasting plasma glucose (mmol/L) |  | *p = .947* | | *p = .539* | | *p = .887* | | *p = .379* | |
|  | Men |  | 0.00 (-0.02, 0.03) |  | 0.01 (-0.03, 0.05) |  | 0.00 (-0.03, 0.04) |  | 0.00 (-0.04, 0.03) |
|  | Women |  | 0.02 (-0.01, 0.04) |  | 0.00 (-0.04, 0.03) |  | 0.01 (-0.02, 0.03) |  | -0.01 (-0.03, 0.02) |
| Systolic blood pressure (mm Hg) |  | ***p = .014*** | | *p = .967* | | *p = .114* | | *p = .352* | |
|  | Men |  | -0.24 (-0.83, 0.34) |  | -0.32 (-1.31, 0.66) |  | 0.79 (0.09, 1.50) |  | 0.00 (-0.83, 0.83) |
|  | Women |  | -0.48 (-1.07, 0.11) |  | -0.18 (-1.32, 0.96) |  | 0.56 (-0.19, 1.32) |  | 0.51 (-0.35, 1.38) |
| Diastolic blood pressure (mm Hg) |  | *p = .217* | | *p = .808* | | *p = .507* | | *p = .665* | |
|  | Men |  | -0.31 (-0.70, 0.09) |  | -0.11 (-0.74, 0.51) |  | 0.45 (-0.06, 0.96) |  | -0.22 (-0.87, 0.43) |
|  | Women |  | -0.33 (-0.74, 0.08) |  | -0.03 (-0.71, 0.66) |  | 0.37 (-0.10, 0.83) |  | -0.12 (-0.56, 0.31) |
| Triglycerides (mmol/L) |  | ***p = < .001*** | | *p = .959* | | ***p = .006*** | | *p = .733* | |
|  | Men |  | 0.03 (0.00, 0.07) |  | -0.02 (-0.07, 0.03) |  | 0.00 (-0.05, 0.04) |  | -0.02 (-0.06, 0.02) |
|  | Women |  | **-0.04 (-0.06, -0.01)** |  | 0.00 (-0.03, 0.04) |  | **0.03 (0.00, 0.06)** |  | 0.02 (-0.01, 0.04) |
| HDL-cholesterol (mmol/L) |  | *p = .193* | | *p = .382* | | *p = .838* | | *p = .588* | |
|  | Men |  | 0.00 (-0.01, 0.01) |  | 0.00 (-0.01, 0.01) |  | -0.01 (-0.02, 0.00) |  | -0.01 (-0.02, 0.01) |
|  | Women |  | 0.01 (-0.01, 0.02) |  | 0.00 (-0.02, 0.03) |  | -0.01 (-0.03, 0.00) |  | 0.00 (-0.01, 0.02) |
| LDL/HDL ratio (mmol/L) |  | ***p = .011*** | | *p = .401* | | *p = .518* | | *p = .366* | |
|  | Men |  | 0.01 (-0.02, 0.04) |  | 0.01 (-0.03, 0.05) |  | 0.02 (-0.02, 0.06) |  | 0.03 (-0.01, 0.07) |
|  | Women |  | -0.01 (-0.04, 0.01) |  | 0.00 (-0.04, 0.04) |  | 0.01 (-0.02, 0.05) |  | 0.01 (-0.02, 0.05) |
| BMI (kg/m^2^)† |  | *p = .063* | | *p = .348* | | *p = .062* | | *p = .099* | |
|  | Men |  | -0.04 (-0.19, 0.12) |  | 0.14 (-0.11, 0.38) |  | 0.05 (-0.15, 0.25) |  | 0.20 (-0.04, 0.44) |
|  | Women |  | -0.27 (-0.49, -0.05) |  | -0.10 (-0.46, 0.26) |  | 0.13 (-0.09, 0.35) |  | 0.50 (0.12, 0.88) |
| Waist circumference (cm)† |  | ***p = .034*** | | *p = .547* | | *p = .171* | | *p = .123* | |
|  | Men |  | -0.06 (-0.49, 0.37) |  | 0.36 (-0.34, 1.05) |  | 0.16 (-0.36, 0.69) |  | 0.33 (-0.37, 1.03) |
|  | Women |  | **-0.74 (-1.15, -0.33)** |  | -0.02 (-0.97, 0.93) |  | 0.39 (-0.05, 0.83) |  | 1.03 (0.32, 1.74) |
| Clustered cardiometabolic risk† |  | ***p = < .001*** | | *p = .407* | | ***p = .010*** | | *p = .277* | |
|  | Men |  | 0.00 (-0.02, 0.02) |  | 0.01 (-0.03, 0.04) |  | 0.02 (0.00, 0.05) |  | 0.01 (-0.02, 0.04) |
|  | Women |  | **-0.04 (-0.06, -0.02)** |  | 0.00 (-0.04, 0.03) |  | **0.04 (0.02, 0.06)** |  | 0.04 (0.01, 0.06) |

*p*-values denote exposure*gender interaction

**Bold** typeface indicates statistical significance, but only if the interaction term is also significant for the stratified models (*p* < 0.05).

All models are adjusted for total sitting time and confounding variables.

†No additional adjustment for waist circumference.
