# Supplementary figures and images for: Associations of context-specific sitting time with markers of cardiometabolic risk in Australian adults
Source: Int J Behav Nutr Phys Act. 2018 Nov 20;15:114. doi: 10.1186/s12966-018-0748-3 (PMC6245709; doi:10.1186/s12966-018-0748-3)

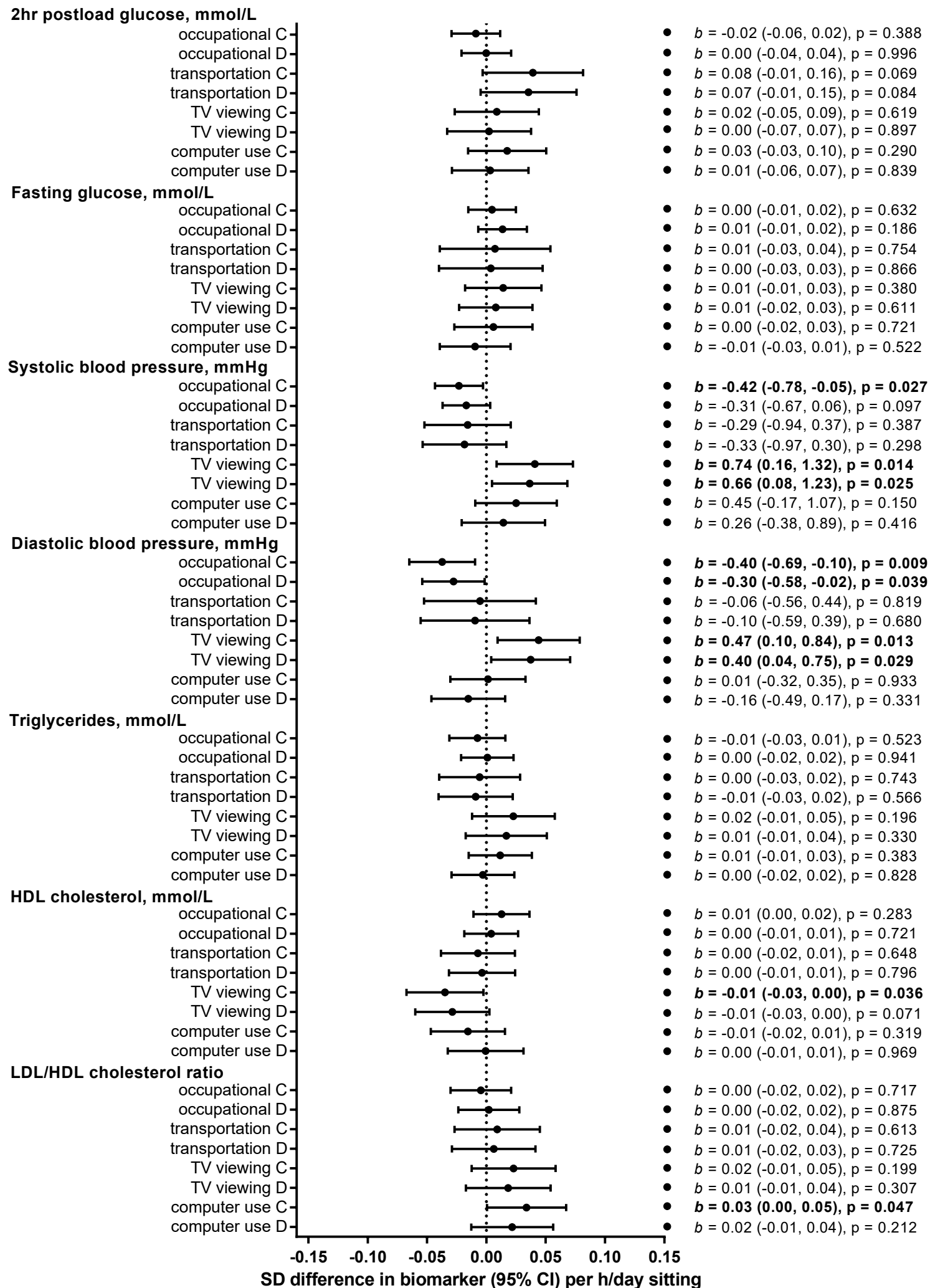

Supplement: Supplementary file 4 — Figure S9. Associations (95% CI) of context-specific sitting time (h/day) with cardiometabolic risk biomarkers, adjusted for potential confounders, total sitting time and further for waist circumference. Associations are plotted standardised (β) and shown in text unstandardised (b). (PDF 66 kb) [file 12966_2018_748_MOESM4_ESM.pdf]
